# A national survey on current state and development needs of clinical and academic emergency medicine in China

**DOI:** 10.1186/s12909-024-05226-3

**Published:** 2024-03-04

**Authors:** Lanfang du, Yan Li, Zhenjie wang, Guoqiang Zhang, Xiaohui Chen, Yingping Tian, Changju Zhu, Jinsong Zhang, Lidong Wu, Peiwu Li, Yuguo Chen, Bing Ji, Shuming Pan, Jun Zeng, Yanfen Chai, Yesai Mu, Mao Zhang, Yu Ma, Chuanzhu Lv, Qingbian Ma

**Affiliations:** 1 Emergency department, Peking university third hospital, 100191 Beijing, China; 2 Emergency department, The first affiliated hospital of Bengbu medical college, 233004 Bengbu, China; 3 Emergency department, 100029 Beijing, China; 4 Emergency department, Guangzhou medical university, 510260 Guangzhou, China; 5 Emergency department, the second hospital of Hebei medical hospital, 050000 Shijiazhuang, China; 6 Emergency department, the first affiliated hospital of Zhengzhou university, 450052 Zhengzhou, China; 7 Emergency department, Jiangsu province hospital, 210029 Nanjing, China; 8 Emergency department, the second affiliated hospital of Nanchang university, 330006 Nanchang, China; 9 Emergency department, Lanzhou university second hospital, 730030 Lanzhou, China; 10 Emergency Department, Qilu Hospital of Shandong University, 250012 Jinan, China; 11 Emergency Department, First hospital of Shanxi medical university, 030001 Taiyuan, China; 12 Emergency Department, Xinhua hospital, Shanghai jiao tong university school of medicine, 200092 Shanghai, China; 13 Emergency Department, Sichuan academy of medical science · Sichuan provincial people’ s hospital, 610072 Chengdu, China; 14 Emergency Department, Tianjin Medical University General Hospital, 300052 Tianjin, China; 15 Emergency Department, Xinjiang Uiger manucipal people’s hospital, 830001 Wulumuqi, China; 16 Emergency Department, The second affiliated hospital Zhejiang university school of medicine, 310009 Hangzhou, China; 17 Chongqing University Central Hospital, Chongqing Emergency Medical Center, 630014 Chongqing, China

**Keywords:** Emergency medicine, Academic emergency medicine, Emergency department, Specialty characteristics, Medical education

## Abstract

**Background:**

To characterize the current state of emergency medicine (EM) and the requirements for advancing EM clinical practice, education and research in China.

**Methods:**

An anonymous electronic survey was conducted by Chinese Society of Emergency Medicine during September to October 2021. The survey contained 30 questions divided into 2 sections: the current state of EM development and the requirements for EM growth.

**Results:**

722 hospitals were included, of 487 were Level III and 235 were Level II hospitals. We found that after 40 years of development, EM had established a mature disciplinary system and refined sub-specialties including critical care, cardiopulmonary resuscitation, toxicology, disaster and emergency rescue. In Level III hospitals, 70.8% of EDs were standardized training centers for EM residents, but master’s degree program, Doctor Degree program and post-doctoral degree program was approved in only 37.8%, 8.4% and 2.9% of EDs respectively and postgraduate curriculum was available in 1/4 of EDs. Only 8% have national or provincial key laboratories. In addition to advance clinical practice, there was also a high demand to improve teaching and research capacities, mainly focusing on literature review, research design and delivery, paper writing, residency training.

**Conclusions:**

EM has built a mature discipline system and refined sub-specialties in China. Teaching and research developed parallel with clinical practice. However, there was still a lack of EM master’s and doctoral programs and research capacities need to be improved. More outstanding clinical and academic training should be provided to promote the rapid growth of EM in China.

Emergency medicine (EM) was a relatively young specialty in the world. In response to increase in emergency visit and the need for high-quality emergency care, EM had developed rapidly in recent decades and continued to expand globally [1]. The emergency department(ED) was the core of the emergency medical service system, and it was a professional department dealing with acute and critical diseases, public emergencies, collective poisoning events, natural disasters, and mass trauma events. The development of EM had been reported worldwide and had been evaluated from many perspectives [2–4]. In China, EM was officially recognized as an independent specialty in mid-1980s and had made great progress after 40 years of hard work [5]. However, to our knowledge, most of the studies on development of EM in China were reviews or editorials and most of them only paid attention to one perspective [5]. Original research on systematic evaluation of current EM state was limited. Forty years of our history in advancing EM will surely provide EM colleagues from all over the world with valuable experiences. Also it was very important to understand the demands for further development of the specialty.

In this study, we conducted a national survey to quantify and describe the emergency visits, sub-special characters, research and teaching capacities, national and international platforms need to be built for advancing EM in China, which mainly based on hospital EDs. Our findings will provide practical suggestions and guidance for improving the quality of emergency care and help the world better understand the EM in China.

## Methods

### Study design

**Fig. 1:**
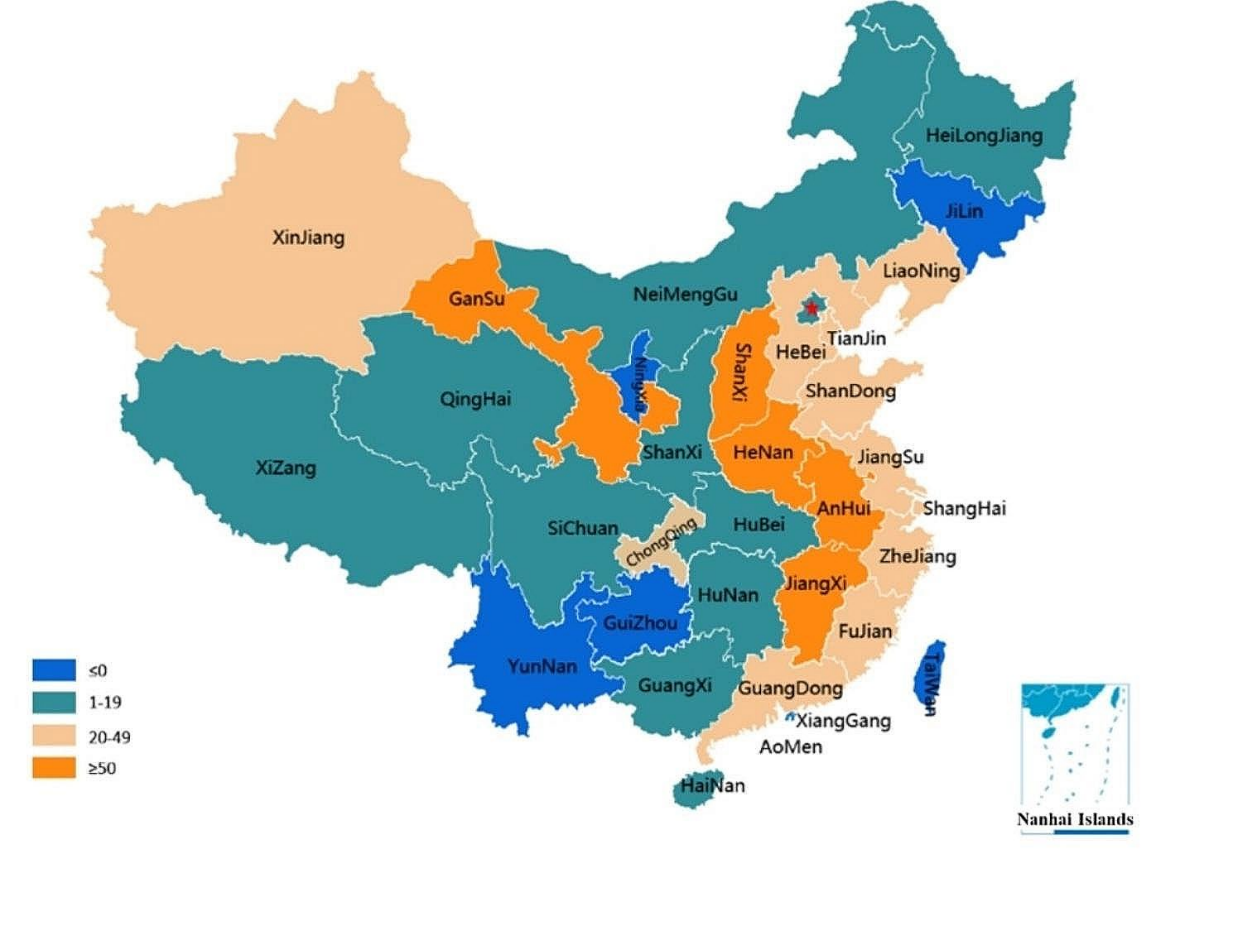
The numbers of surveyed hospitals in each province of China

This study was a descriptive, cross-sectional survey conducted by Chinese Society of Emergency Medicine (CSEM). The CSEM is a national society established in 1987 to ensure high quality care by setting standards of care and providing expert guidance on policy to relevant bodies on matters relating to EM. It consists of 68 members from all over China with good geographical representation, 31 of whom serve as chairpersons of the provincial emergency societies in total 31 provinces of Chinese mainland. In order to get a high response rate and high quality control, multistage convenience sampling was used. First nearly all the membership of CSEM participated the survey on behalf of their hospital. Then the 31 provincial chairpersons send questionnaires to members of their responsible provincial emergency societies. Each hospital was reminded 5 times and they volunteered to take part in the study. Only director of emergency department was responsible for completing the questionnaire. Finally, 722 hospitals were selected. The numbers of surveyed hospitals in each province were shown in Fig. 1.

In China, Hospitals are classified by the National Health and Family Planning Commission of the People’s Republic of China as follows: Level III hospitals refer to medical centers or tertiary hospitals that have independent emergency departments (EDs) and are also teaching hospitals, mainly responsible for comprehensive undergraduate, graduate and postgraduate medical education; Level II hospitals are regional hospitals which have independent ED but are not teaching hospitals, mainly responsible for clinical practice training in medical college and continuing education in primary hospitals; and Level I hospitals, also called community hospitals, do not have EDs, mainly focus on providing medical service for community. In this study, 487 were Level III and 235 were Level II hospitals.

The CSEM leadership designed the survey tool which consisted of 30 questions covering categories including the current state of EM and the requirements for advancing practice, education and research of EM. A link to the electronic survey was distributed during September 2021. Data collection ended in October 2021. All protocols were approved by Peking university third hospital.

### Statistical method

The data were analyzed by SPSS 20.0. Quantitative variables were expressed as mean (standard deviation) when following a Gaussian distribution or median (inter quartile range 25∼75%) otherwise. Categorical variables were expressed as frequencies. Data was tested for normality using the Shapiro-Wilk Normality Test.

## Results

Among 722 hospitals, university affiliated hospitals accounted for 33.5% (242/722), university teaching hospitals were 17.3% (125/722) and other public hospitals were 49.2% (355/722).

## Current status of EM in China

**Fig. 2:**
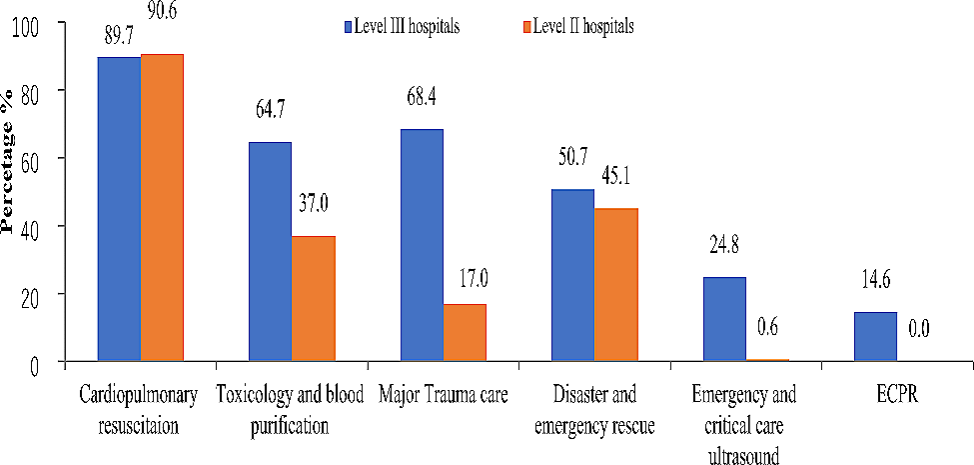
**Clinical sub-special characteristics of emergency departments in China** **(**Answer to the question: what are the sub-special characteristics of your department**)**

As shown in Table 1, annual emergency visits were 60,000 and 20,000 in Level III and Level II hospital respectively before COVID-19. In Level III hospitals, 70.8% of EDs were standardized training centers for EM residents, but master’s degree program, Doctor Degree program and post-doctoral degree program was approved in only 37.8%, 8.4% and 2.9% EDs respectively, postgraduate curriculum was available in 1/4 of EDs. The specialty characteristics were shown in Fig. 2. Most EDs in Level III or Level II hospitals were characterized by subspecialties of critical care, cardiopulmonary resuscitation (CPR), toxicology, disaster and emergency rescue. EDs in Level III hospitals did better in ECPR, major trauma care, emergency and critical care ultrasound than that in Level II hospitals.

**Table 1:** The current status of EM in China

Items	Level III hospitals (*n* = 487)	Level II hospitals (*n* = 235)
Total Beds in ED (n)	38[20,60]	17[10,34]
Annual emergency visits before COVID-19 (ten thousand)	6[3.2,12]	2[1,3.5]
Number of emergency physicians (n)	24[16,35]	10[6,13]
EM Master’s Degree program, n(%)	155(31.8)	0(0%)
EM Doctoral Degree program, n(%)	41(8.4)	0(0%)
EM Post-doctoral Degree program, n(%)	14(2.9)	0(0%)
EM postgraduate curriculum had been offered, n (%)	127(26.1)	0(0%)
National or provincial EM key laboratories, n (%)	38(7.8)	0(0%)
Standardized training centers for EM residents, n (%)	349(70.8)	31(13.2)

ED: emergency department; EM: emergency medicine

## Requirements for advancing clinical practice, education and research of EM in China

**Fig. 3:**
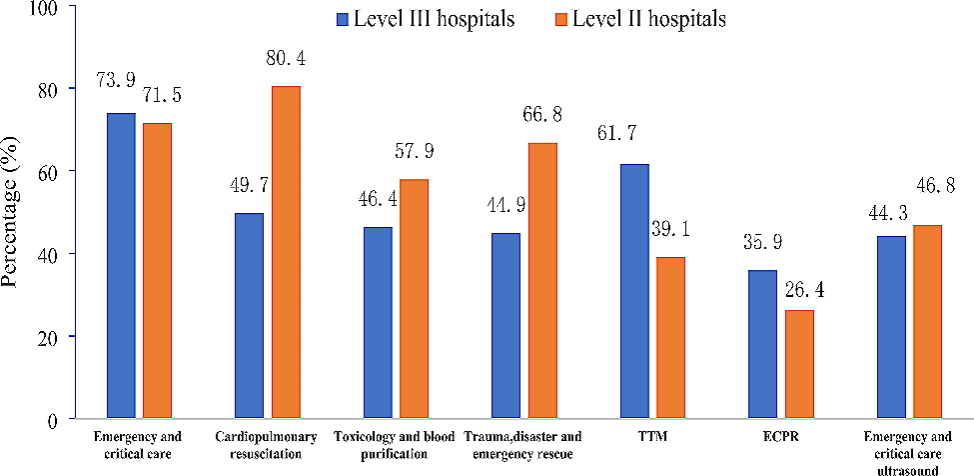
**The academic platforms for clinical exchange need to be built in emergency medicine in China** (Answer to the question: which of the following academic exchange platforms should be built based on clinical needs) TTM: target temperature management; ECPR: Extracorporeal cardiopulmonary resuscitation

The platforms need to be built for improving clinical practice of EM were shown Fig. 3. There was a strong demand for improving clinical practice of EDs in Level II hospitals, mainly focused on emergency and critical care, cardiopulmonary resuscitation, trauma, disaster and emergency rescue, toxicology and point-of-care ultrasonography for emergency and critical patients. The EDs in Level III hospitals wanted to learn more about advanced critical techniques, including target temperature management (TTM), extracorporeal CPR (ECPR).

**Fig. 4:**
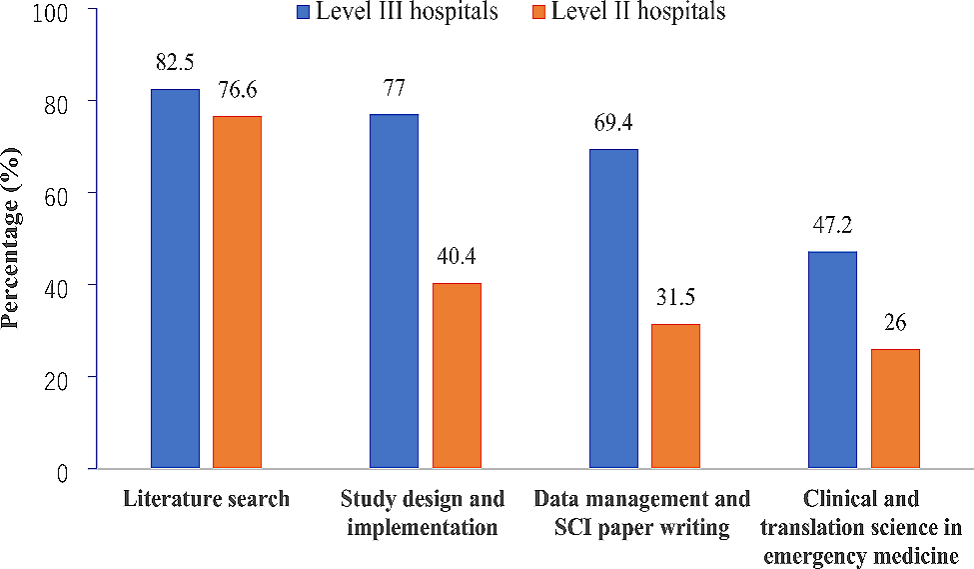
**Demands for improving research capacities in emergency medicine in China** **(**Answer to the question: which of the following academic exchange platforms should be built to improve research capacities**)**

**Fig. 5:**
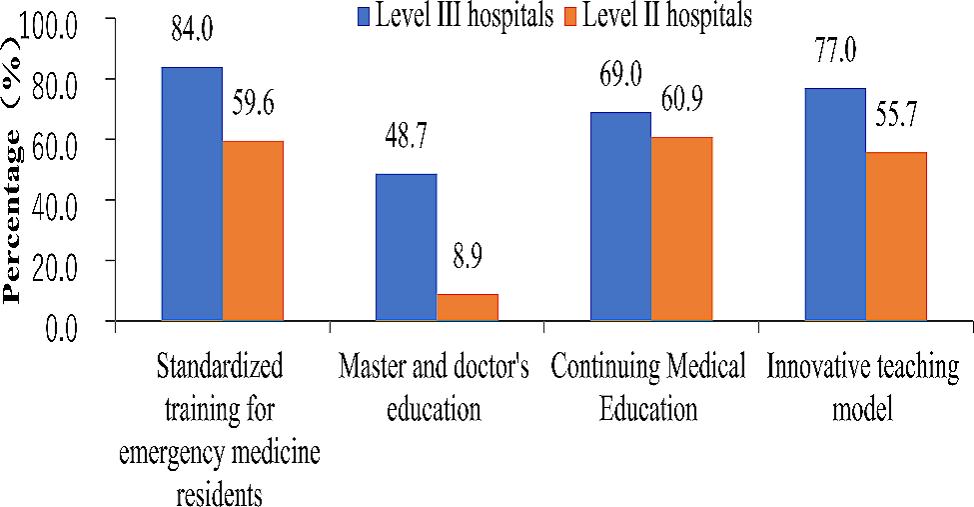
**Demands for advancing teaching and education in emergency medicine in China** **(**Answer to the question: which of the following academic exchange platforms should be built to improve teaching capacities**)**

As shown in Fig. 4, emergency physicians mainly wanted to improve their research ability in literature search, study design, data management and paper writing, especially in level III hospitals. The good news is that nearly 50% of EDs in Level III hospitals were interested in clinical and translation science. As shown in Fig. 5, the standardized training for EM residents was the most concerned topic in emergency education in both Level III and Level II hospitals, following by innovative teaching model and continuing medical education. Compared with EDs in Level II hospitals, EDs in Level III hospitals need more discussions on master’s and doctoral education.

## Discussion

To our knowledge, this is the first comprehensive national survey on the current EM status and requirements for further growth in China. We found that EM had established a relatively mature disciplinary system and refined sub-specialties including critical care, cardiopulmonary resuscitation, toxicology, trauma, disaster and emergency rescue. The EM teaching and education system was initially established, and the emergency residents standardized training had been implemented almost all over the country. However the graduate EM education was underdeveloped, only a few EDs in Level III hospitals had master’s or doctoral degree programs. In addition to improve emergency clinical practice, there was also a strong need to advance research and teaching capacities.

In China, The first ED was founded in 1983 and EM was recognized as an independent specialty in 1986. It kept developing in China over the past 40 years as a response to the increase of emergency visits and the requirement for high-quality care and experienced three stages in China, including primary period, stable developing period and rapid developing period [6, 7]. EDs were gradually established in all Level III and Level II hospitals in primary period(1980–2009). Standards were set for emergency physicians’ access, emergency care quality, EDs’ infrastructure in stable developing period(2009–2017), indicating transformation of the ED from emergency room to a professional discipline. It entered rapid developing period since 2018. In this period, emergency resources were integrated to improve national emergency care quality and five centers(Chest pain center, Stroke center, Trauma center, Maternal critical care center, Pediatric and Neonatal Critical Care center) were established in nearly all the Level III and Level II hospitals [8].

However, the shortage should be noticed at the same time. As a safe, timely diagnostic device [9], point-of-care ultrasound(POCUS) was standard care of critically ill patients as early as 10 years ago and POCUS training in EM residency programs was prevalent in developed countries [10–12]. However we found that it was not very popular in China. The reason may be related to lack of standardized training program and excessive reliance on official ultrasound or echocardiogram provided by cardiologists. The application of ECPR increased rapidly in China during the past 10 years. A national survey in 2017 showed only 2.5% of Level III hospitals had performed ECPR and our study found that 14.6% of Level III hospitals refined ECPR as specialty characteristics [13].

As the clinical specialty advanced, so did the EM research. According to Lee, the number of articles published in international EM journals increased substantially in the past 20 years in China [14]. However, it was still much less than that in the first countries to recognize EM as a specialty, especially lack of high-quality research [15]. This may be associated with work overload, shortage of basic research and lack of national emergency and critical disease cohort. In addition to increase funding for EM research, emergency research network should be established and national or local research engagement events for EM trainees should be held to improve research capacities [16–18]. Our survey revealed that there was a strong desire for research training focus on literature search, study design, data management and paper writing, especially in Level III hospitals.

Standardized residency training was the most concerned topic in EM education. EM residency training and fellowship programs began in the late 1990s but was not formalized until the end of 2013 in China, almost 20 years later than in USA [19]. Residency program standardized to 36 months of total training with a minimum 18 months in ED and ICU [5]. Fellowship programs need 24 months of total training mainly in ED and ICU. All medical students were required to attend residency training to become a qualified EM resident, and fellowship programs to become a qualified EM attending physician in China. As shown in our survey, 70.8% of EDs in Level III hospitals were residency training centers. As similarly in USA, the programs taught residents to master many diagnostic, procedural, and interpersonal skills required of EM physicians [20]. Also there were many fellowship programs in developed countries, including fellowship in emergency critical care, emergency medical education, emergency ultrasound, emergency department administration and so on [21]. At present we mainly focus on fellowship in emergency critical care.

In China the first EM master’s and doctoral degree program was established in 1985 and 2003 and the EM graduate education system had been initially established after nearly 40 years of development. 37.8% EDs in Level III hospitals had master’s degree programs. As in the USA, there was huge gap between the supply and demand of EM graduate students [22]. The licensed emergency physicians raised triple from 20,058 to 59,409 during the past 20 years, but most of them were physicians from specialties other than EM [23]. We should also noticed that only 8.4% had doctoral degree programs and 2.9% had post-doctoral degree programs, indicating serious shortage of EM professionals a high degree of academic and clinical competence. So we should further optimize the EM education system. Ideally, EM should be Integrated into core curriculum of undergraduate medical education and further efforts should be made to improve graduate education.

## Limitations

In our study, convenience sampling was used in our study to get a high response rate. It was very difficult to conduct a randomized observational survey. Despite surveyed hospitals were geographically well distributed in this study, it did have disadvantages of convenience sampling. Although our study was an online survey, pandemic might lower the response rate due to work overload caused by COVID-19. Also the pandemic influenced some of the information gathered through the survey, they might pay more attention to emergency rescue and innovative teaching methods in this context.

## Conclusions

EM had refined sub-specialties including critical care, cardiopulmonary resuscitation, toxicology, trauma, disaster and emergency rescue. Teaching and research developed parallel with clinical practice. However, there was still a lack of EM master’s and doctoral programs and research capacities need to be improved. In the future, more outstanding clinical and academic training should be provided to promote the rapid growth of EM in China.

## Data Availability

The datasets analysed during the current study are available from the corresponding author on reasonable request.

## Abbreviations

EM: emergency medicine
ED: emergency department
CSEM: Chinese Society of Emergency Medicine
CPR: cardiopulmonary resuscitation
TTM: target temperature management
ECPR: extracorporeal CPR
