# Correction: A national survey on current state and development needs of clinical and academic emergency medicine in China

**DOI:** 10.1186/s12909-024-05387-1

**Published:** 2024-04-17

**Authors:** Lanfang du, Yan Li, Zhenjie wang, Guoqiang Zhang, Xiaohui Chen, Yingping Tian, Changju Zhu, Jinsong Zhang, Lidong Wu, Peiwu Li, Yuguo Chen, Bing Ji, Shuming Pan, Jun Zeng, Yanfen Chai, Yesai Mu, Mao Zhang, Yu Ma, Chuanzhu Lv, Qingbian Ma

**Affiliations:** 1 Emergency department, Peking university third hospital, 100191 Beijing, China; 2 Emergency department, The first affiliated hospital of Bengbu medical college, 233004 Bengbu, China; 3 Emergency department, 100029 Beijing, China; 4 Emergency department, Guangzhou medical university, 510260 Guangzhou, China; 5 Emergency department, the second hospital of Hebei medical hospital, 050000 Shijiazhuang, China; 6 Emergency department, the first affiliated hospital of Zhengzhou university, 450052 Zhengzhou, China; 7 Emergency department, Jiangsu province hospital, 210029 Nanjing, China; 8 Emergency department, the second affiliated hospital of Nanchang university, 330006 Nanchang, China; 9 Emergency department, Lanzhou university second hospital, 730030 Lanzhou, China; 10 Emergency Department, Qilu Hospital of Shandong University, 250012 Jinan, China; 11 Emergency Department, First hospital of Shanxi medical university, 030001 Taiyuan, China; 12 Emergency Department, Xinhua hospital, Shanghai jiao tong university school of medicine, 200092 Shanghai, China; 13 Emergency Department, Sichuan academy of medical science · Sichuan provincial people’ s hospital, 610072 Chengdu, China; 14 Emergency Department, Tianjin Medical University General Hospital, 300052 Tianjin, China; 15 Emergency Department, Xinjiang Uiger manucipal people’s hospital, 830001 Wulumuqi, China; 16 Emergency Department, The second affiliated hospital Zhejiang university school of medicine, 310009 Hangzhou, China; 17 University Central Hospital, Chongqing Emergency Medical Center, 630014 Chongqing, China

**Correction: BMC Medical Education (2024) 24:229** 10.1186/s12909-024-05226-3

Following publication of the original article [1], we were notified that the Correspondence details were missed.

The original article has been corrected.
